# Toward biophysical markers of depression vulnerability

**DOI:** 10.3389/fpsyt.2022.938694

**Published:** 2022-10-18

**Authors:** D. A. Pinotsis, S. Fitzgerald, C. See, A. Sementsova, A. S. Widge

**Affiliations:** ^1^Centre for Mathematical Neuroscience and Psychology, Department of Psychology, City, University of London, London, United Kingdom; ^2^The Picower Institute for Learning and Memory, Department of Brain and Cognitive Sciences, Massachusetts Institute of Technology, Cambridge, MA, United States; ^3^Department of Computer Science, City, University of London, London, United Kingdom; ^4^Department of Psychiatry and Behavioral Sciences, University of Minnesota, Minneapolis, MN, United States

**Keywords:** depression, dynamic causal modeling (DCM), biomarkers, event-related potentials (ERPs), machine learning

## Abstract

A major difficulty with treating psychiatric disorders is their heterogeneity: different neural causes can lead to the same phenotype. To address this, we propose describing the underlying pathophysiology in terms of interpretable, biophysical parameters of a neural model derived from the electroencephalogram. We analyzed data from a small patient cohort of patients with depression and controls. Using DCM, we constructed biophysical models that describe neural dynamics in a cortical network activated during a task that is used to assess depression state. We show that biophysical model parameters are biomarkers, that is, variables that allow subtyping of depression at a biological level. They yield a low dimensional, interpretable feature space that allowed description of differences between individual patients with depressive symptoms. They could capture internal heterogeneity/variance of depression state and achieve significantly better classification than commonly used EEG features. Our work is a proof of concept that a combination of biophysical models and machine learning may outperform earlier approaches based on classical statistics and raw brain data.

## Introduction

Depression affects roughly one in six people (1), and its prevalence may be increasing (2). A major difficulty with treating depression, and psychiatric disorders in general, is their heterogeneity: a clinical phenotype or classification can arise from multiple different neural causes (3, 4). To address this heterogeneity, we propose describing depression in terms of interpretable, biophysical parameters of a neural model, derived from the electroencephalogram (EEG). These parameters may serve as biomarkers, variables that allow subtyping of depression at a biological level. They can be thought of as latent variables that may capture individual differences between patients. As a proof of concept, we show that this idea works even in a small patient cohort.

Several studies have used EEG data to identify potential biomarkers for psychiatric disorders (5–8). These studies emphasized EEG features: components of event-related potentials (ERPs) or oscillatory responses, not biophysical parameters. For example, multiple papers report smaller N1 amplitudes in depression (6, 9, 10). However, the results from these studies are difficult to connect back to biology: The data features (e.g., ERPs, oscillatory responses, and resting state activity) do not directly map back to brain structures or to physiologic changes. There are exceptions, e.g., the loudness dependence of the auditory evoked potential (11–13), but in general these analyses are mainly phenomenological. They also fail to consider depression’s internal heterogeneity, which limits generalizability of the derived biomarkers. A recent meta-analysis suggested that no EEG marker had reliable clinical utility (14), although newer work has tried to address this (15, 16).

One approach to overcoming depression’s heterogeneity might be to shift the level of analysis. For instance, source localization techniques can interpret scalp phenomena in terms of their underlying cortical generators (8, 17). Still these methods emphasize waves/patterns in the electrical activity whose neural basis remains unclear. A deeper level of analysis more grounded in cellular physiology may be possible when using biophysical models. This is the approach we take here. Their parameters describe the neurobiology or neural populations (e.g., synaptic time constants, intrinsic, and extrinsic connectivity) that give rise to the scalp-recorded patterns. They capture important developmental, structural and functional properties of cortical sources. Synaptic time constants are important for determining the EEG signal (18). Intrinsic and extrinsic connectivity go through characteristic changes throughout the development of the brain and can exhibit differences with age or in the presence of a disease (19). For instance, we and others have used biophysical models to analyze data from patients with neurological diseases recorded using M/EEG and fMRI (20–24).

Here, we constructed biophysical models using Dynamic Causal Modelling (DCM). These describe the cortical network activated during a cognitive conflict task that activates depression-relevant brain areas (25–27). Our model transforms high-dimensional EEG data onto a mechanistically interpretable feature space (20); in which, we show below that we can better measure depression’s internal heterogeneity. We present a proof of concept for the following idea: that biophysical model parameters yield a low dimensional, interpretable feature space. As a result of that better capture of internal heterogeneity/variance, model-derived features achieved significantly better classification than manifest EEG features. Our work shows that a combination of biophysical models and machine learning may be an alternative to earlier approaches based on classical statistics and raw brain data.

## Materials and methods

### Dataset

The dataset included 15 psychiatric patients who reported current or past depressive symptoms and 34 non-diagnosed controls. Importantly, this dataset was not limited to patients diagnosed with unipolar depression, but included bipolar and unspecified depression. We considered this a better demonstration of our approach to heterogeneity. This is a secondary analysis of a cohort collected in a previous study (28). For details of the EEG recordings, see C Methods.

**FIGURE 1:**
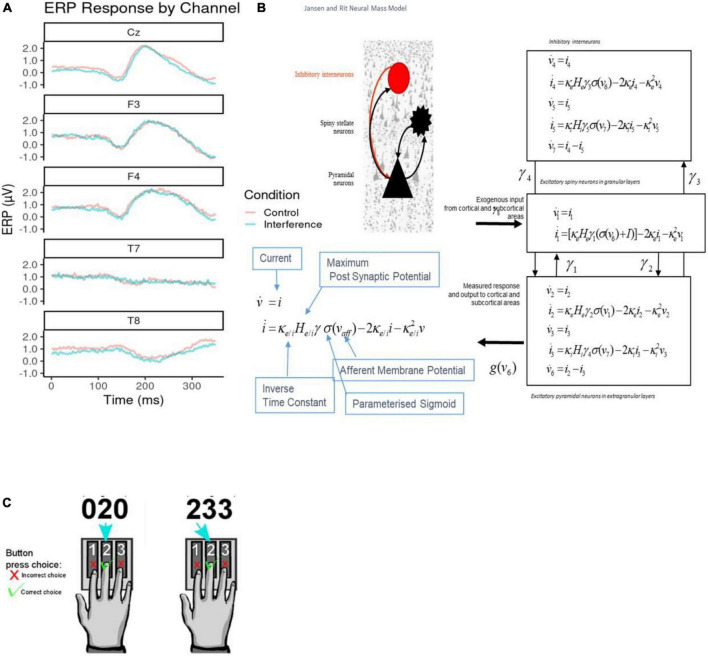
**(A)** Sample average electroencephalogram (EEG) responses. These are shown per channel retained for classification analysis. Both control (red) and interference condition (blue) averages are plotted across the 350°ms response time post stimulus onset. Channels Cz, F3, and F4 show typical event-related potentials (ERP) amplitudes, while T7 and T8 show flat EEG responses across the duration. **(B)** Jansen and Rit Model. The schematic diagram summarizes the evolution equations that specify a Jansen and Rit (JR) neural mass model of a single source. This model contains three populations, each loosely associated with a specific cortical sub-population or layer: pyramidal and spiny stellate neurons and inhibitory interneurons. Second-order differential equations mediate a linear convolution of presynaptic activity to produce postsynaptic depolarization. This depolarization gives rise to firing rates within each sub-population that provides inputs to other populations. The operations are captured by the equations shown on the right-hand side, which are explained in the bottom-left comer inset that includes the parameters appearing in these equations and then definitions. For a thorough discussion of these equations, see the study by (26). **(C)** Multiple Source Interference Task (MSIT). The task requires participants to report on a presented stimulus by using their index, middle or ring finger to press three buttons corresponding to numbers 1, 2, and 3 respectively. The stimulus appears on a screen displaying three numbers; one number (the target) is different from the other two (distractors). The participant identifies the target by pressing the corresponding buttons. There are two task conditions, control, and interference. During control trials, the distractor numbers are zeros and the location of the target number is aligned with its corresponding button. In interference trials, the distractors are non-zero numbers, and the target is in a location misaligned with that of the button.

Electroencephalogram (EEGs) were collected as participants performed the Multi-Source Interference Task (MSIT), see Figure 1C (29). MSIT has been validated to produce robust cortical activations at the single-participant level, in both fMRI (29) and EEG (30) studies, and is used for assessing depression state. For more details regarding the task and dataset, see Supplementary methods.

### Event-related potentials analysis

We emphasized a Positive Potential signal, defined as evoked responses between 250 and 350°ms after event onset. Positive Potential components were extracted from 70 EEG channels (average ERPs over participants are included in Figure 1A; see also Supplementary Figure 1). Previous MSIT studies found differences in similar Positive Potential components between task conditions (31, 32). These early Positive Potentials are a common signature of conflict and cognitive control, and arise when incongruent stimuli are processed (33, 34). We used Positive Potential peak amplitude and latency as EEG classification features (see Class Balancing and Model Training section below). To test for the effect of conditions (interference vs. control), Wilcoxon tests were conducted on each channel to compare the peak amplitude and latencies. Latency exhibited significant differences, see Supplementary Table 1. See also Supplementary methods for more details on the ERP extraction pipeline.

### Dynamic causal modeling

We used Dynamic Causal Modelling (DCM) (20–22, 35–40) to infer processes at the neuronal level from scalp EEG measurements (2). We characterized changes of intrinsic (within area) and extrinsic (between area) connections across task conditions and between individuals. We assessed whether information flow changed in the same way (top-down, bottom-up or both) between the two task conditions across all participants. We here used DCM for Evoked Responses and Jansen and Rit (JR) mass model (Figure 1B). JR models can predict both evoked and induced responses and have been used in theoretical and experimental studies (27, 41–44). DCM was implemented using SPM12. For more details about DCM, see Supplementary methods.

### Functional network

**FIGURE 2:**
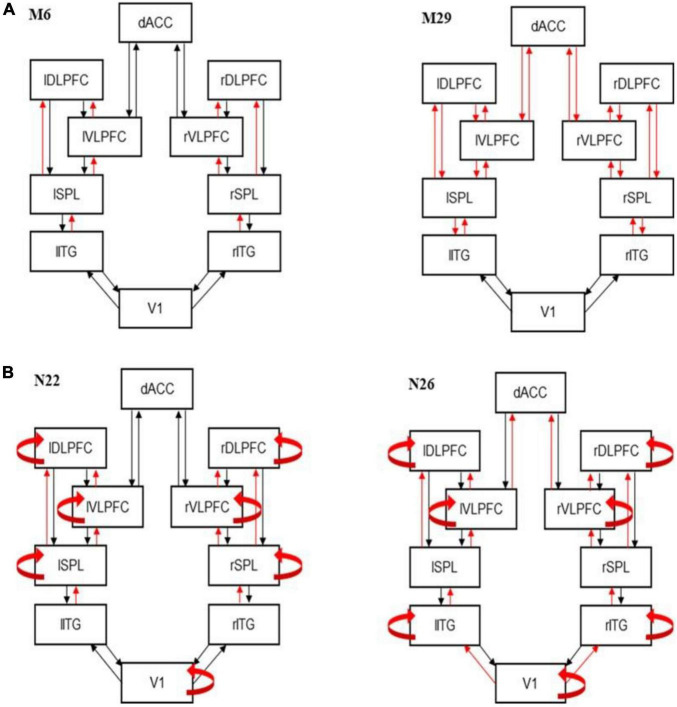
Best fitting models. **(A)** Dynamic causal modeling (DCM) best fitting model M6 (left) and runner up model M29 (right). Model M6 includes changes in forward connections at all levels except VI. The runner up (M29) is very similar. It also includes the corresponding feedback connections on top of the forward connections included in M6. **(B)** Best fitting DCM models showing modulations of intrinsic connections for controls (N22; left) and patients (N26; right).

The functional network modeled with DCM can be seen in Figure 2 (cf. model M1 in top left corner, all other models include the same network and assume changes in different connections, explained below). This network is comprised of areas activated during the MSIT (29, 45). The network included sensory, temporal, parietal, dorsal and ventral frontal areas, and ACC: V1, dACC and the following areas in both hemispheres: ITG, SPL, vlPFC, dlPFC. Changes in functional connectivity within this network were observed, at the group level, in patients with depression (46–49). For details about the coordinates of these areas, and why we chose this network and no other areas, see Supplementary methods. We used DCM parameter estimates as data features for classification and clustering.

### Dynamic causal modeling parameters

Dynamic causal modeling (DCM) parameter estimates were obtained by fitting ERPs, i.e., Positive Potentials evoked during the MSIT task. We fitted ERP recordings from different participants, patients, and controls. We thus obtained DCM parameter estimates. Noise or heterogeneity in the scalp-level recordings might arise from a small number of disruptions in the underlying network. After fitting, variability in ERP recordings leads to variability in the biophysical model (DCM) parameter estimates across participants. This, in turn, could describe biotypes or endophenotypes of depression. We hypothesize: (1) If DCM can capture that variability, then DCM-derived model parameters might be more effective than raw ERPs at classifying patients from controls; (2) Clustering of DCM parameters may help identify clusters of endophenotypes. We tested these hypotheses below.

Dynamic causal modeling (DCM) parameters were obtained after fitting data from individual subjects. These included the following parameters: extrinsic connectivity, *A* (12 × 2 = 24 parameters), differences in extrinsic connectivity between MSIT conditions, *B* (24 parameters, derived from the model fitting as shown in Results), excitatory and inhibitory receptor density, *G* (2 × 10 = 20 parameters), strength of connections between the three populations of the JR model shown in Figure 1B, *H* (4 parameters; see arrows in Figure 1B), and excitatory and inhibitory synaptic time constants, *T* (20 parameters).

### Model comparison

Because we did not know how connectivity changed between task conditions, we compared several variants of the biophysical model describing the network of Figure 2. We considered a network containing all of our modeled brain regions: V1, ITG, SPL, vlPFC, dlPFC, and dACC. We assumed forward and backward connections between specific areas, as well as lateral connections between homologous areas in the right and left hemispheres. We asked which connections might change between MSIT conditions and considered all possible changes. The alternative model variants differed in the connections that could change. Following (39), we first considered changes of extrinsic connections (i.e., between nodes) only. Then in step 2, changes in intrinsic connections. The first twenty candidate models with extrinsic connections changes that we considered, are shown in Supplementary Figure 2. Overall, the candidate model space comprised 45 models. We describe in detail these 45 models in Supplementary methods. The 45 models included all possible models where forward or backward connections changed between different parts of the brain network. Finding the most likely among these models yielded the extrinsic connections that were modulated during the task. For model comparison, we used an approach known as Bayesian model selection (BMS). This was performed assuming fixed-effects (FFX) (50). BMS fits competing models to EEG data and assesses the most likely model. See Supplementary methods for more details.

We considered variations of the network shown in Figure 2. We assumed changes in intrinsic connections from each node to itself (in addition to changes in extrinsic connections that the winning model above assumed) (39). We thus assumed that intrinsic connections could change at any (combination of) brain areas: V1, ITG, SPL, {vlPFC, dlPFC}, and dACC. We thus compared 32 candidate models in total.

### Classification features

The biophysical parameters of the best DCM model obtained *via* BMS were used as features for patient classification and subtyping.

Two sets of classification features were used. DCM parameters and EEG features. DCM parameters were directly compared to EEG features. The DCM parameters included intrinsic and extrinsic connections that were found to differ between MSIT conditions in both the patient and control DCM fits. This resulted in 92 DCM parameters. These were used as DCM predictors in machine learning classifiers.

To compare the predictive power of the DCM parameter estimates with raw EEG features, we used an equal number of ERP features (51). The full set of potential EEG features included 240 variables (60 EEG Channels x 2 conditions x 2 variables, i.e., ERP peak amplitude and latency differences between the two MSIT conditions). We reduced the number of channels to 23 so that the total number of EEG features was the same as the number of DCM parameters. This reduces bias in the comparison between ERP and DCM feature sets. To choose these 23 channels, we performed permutation testing that assesses the change in prediction error of classification after permuting a feature (52, 53). The ERP features were chosen based on their contribution to a random forest model (constructed without hyperparameter tuning). This “naive” random forest allowed us to select channels with features that were most beneficial in separating classes while still allowing for multiple interaction effects between features. The tradeoff of this method stems from using a reduced number of features with the benefit that they are potentially more meaningful, and easier to interpret.

Critically, the above selection of ERP features, biases the subsequent machine learning analysis against our *a priori* hypothesis that DCM-based features will provide at least equivalent classification and clustering–the DCM analysis considers an unselected set of model parameters, whereas the ERP analysis begins with features already known to have some classification power. The selected channels are included in Supplementary Table 2.

### Class balancing and algorithm training

The dataset was imbalanced between control and patient classes. Only 15 of the 49 participants being patients with depressive symptoms. We implemented Synthetic Minority Over-sampling Technique (SMOTE) to correct for this imbalance (54). For more details, about oversampling, see Supplementary methods. This brought parity to the classes with 34 observations each (patients and controls).

Given the sample size limitations, we used 10-fold cross-validation to train and tune machine learning classifiers (55, 56). See Supplementary Figure 9 for a visual depiction of the sampling strategy and Supplementary methods. The cross-validation was used to train classifiers and assess whether DCM features can better measure depression’s internal heterogeneity, compared to EEG features (56). We compared the performance of different machine learning algorithms to distinguish patients with depression vulnerability from controls in both the EEG and DCM feature sets. The algorithms included Support Vector Machines (SVM) (57), Random Forests (52, 58), and Gradient Boosted Trees (59). Comparing DCM and EEG features using multiple algorithms ensures that conclusions are not sensitive to the specific algorithm used. We also used multiple performance metrics, including *F*-measure (F1-score), and the Matthews’ Correlation Coefficient (MCC) (60). See Supplementary methods for more details.

### Feature importance

The best performing classifier as determined by mean MCC score was used to compute feature importance. Shapley additive explanation (SHAP) values were constructed for predictions on the original data set (49 participants, no SMOTE augmentation) (61). This reveals how efficient the low dimensional space spanned by DCM and EEG classification features is in describing the internal heterogeneity of patients with depressive symptoms. SHAP values were constructed using subsampling of different combinations of input features and attributing a weight representing how much credit features should receive for class prediction. The predictive power of EEG and DCM features was compared directly because the corresponding SHAP values take on the same scale and are predicting the same underlying data.

### Unsupervised clustering

The ten most important features as determined by SHAP values from both the ERP and DCM feature sets were used to construct embedding scores with t-stochastic neighbor embeddings (*t-*SNEs). *t-*SNEs are useful for exploring higher-dimensional data in lower dimensional representations when non-linear relationships exist in the data (62). For more details on t-SNE see Supplementary methods. This provided visualizations of the data that were convenient for assessing subtypes or clusters of patients with depressive symptoms.

Clustering was performed using *k-*means in the three dimensional space obtained by *t-*SNE. *K*-means is an unsupervised machine learning method that groups observations to reduce within-cluster sum squares distances and increase the sum squared distance between cluster centroids (63, 64). *K*-means depends on an *a priori* number of clusters. The optimal cluster number can be found by computing Silhouette scores across candidate values of *k*. Observations which been classified appropriately have a lower mean distance between points within their assigned cluster compared to the mean distance to points in the next-nearest cluster neighbors (65). This ratio is given by Silhouette scores.

## Results

### Dynamic causal modeling

We first asked how information flow changes between the congruent and incongruent condition of the MSIT. We used Bayesian Model Selection (BMS; see Supplementary methods) to find the connectivity pattern between our six modeled areas. We fitted ERP data using a biophysical DCM model (Figure 1B) and scored all possible model variants that corresponded to different subsets of connections that might change between MSIT conditions. Different competing models represented different combinations of modulated forward or backward extrinsic connections (see also Methods). BMS identified the winning model as M6 (*BF* > 3; Figure 2A, see also Supplementary Figure 4). Model M6 includes changes in forward connections between ITG→SPL, SPL→vlPFC, SPL→dlPFC, and vlPFC→dLFPC. The runner up (M29) is very similar to M6 (Figure 2B). It includes the corresponding feedback connections on top of the forward connections included in M6. M29 also includes changes in feedforward and feedback connections between vlPFC, dLFPC, and dACC. Thus, the prominent difference between task conditions was changes in the forward (bottom-up) information flow between sensory processing and associative regions. This corresponds to different processing of sensory input between the two MSIT conditions that gives rise to ERP differences in the first 350°ms after stimulus presentation.

In the second step, BMS was used to test the modulation of the intrinsic connections. In this step, we fixed the extrinsic connections to those shown in the winning model M6 above. We then considered variants of M6, where intrinsic connections (within each brain area) were allowed to change. These variants formed a different model space to the one considered above (Methods and Supplementary Figure 3). BMS identified that variant N22 had the highest evidence (*BF* > 3; Figure 2B, see also Supplementary Figure 5). This included modulated intrinsic connections in V1, SPL, vlPFC, and dlPFC areas. The runner up (N23) is exactly the same as N22 with the additional modulation of dACC intrinsic connections (Supplementary Figure 5). We also performed the same BMS for the patient cohort.

We repeated the earlier analysis, where we fitted the neural mass model to patient data only. We wanted to capture the intrinsic heterogeneity. We first found that model M8 best described the changes in extrinsic connections (Supplementary Figure 6). This included changes in all feedforward connections. The runner up (M41) was similar to the winning model, M8, in that it included changes in the feedback connections too. The difference from M8, was that it did not include changes in feedforward (and feedback) connections between V1 and SPL and vlPFC and dACC. The winning model for patients, M8, was similar to the winning model for the control cohort (M6). The difference between the winning models was that two more brain regions showed modulations of feedforward connections. Patients showed changes in vlPFC to dACC and V1 to ITG, in addition to changes in forward (bottom-up) information flow between the ITG to SPL, SPL to vlPFC and dlPFC, and vlPFC to dlPFC that we had found for controls. However, we did not use this difference for our clustering analyses below. Thus, we do not claim that the additional connection changes that we found for the patient cohort are biomarkers of depression or depressive vulnerability. Rather, the similarities (common connections) between M6 (controls) and M8 (patients) were the inputs to machine learning algorithms together with the biophysical parameters described in the last paragraph of this section below.

In the second step, similar to the analysis above, we identified N26 as the model with highest evidence (*BF* > 3; Figure 2B, see also Supplementary Figure 7). This is very similar to N22 that was the winning model for controls. The only difference between N22 (controls) and N26 (patients) is that N22 includes changes in intrinsic connections in ITG instead of SPL. The runner up (N9) is also very similar and assumes modulations of intrinsic connections in dACC instead of V1. Again, we are not claiming this change to be a biomarker of depression, but as an example of how DCM can identify underlying neurological variability. The clustering/classification analysis used only the intrinsic connections that were common to N26 and N22.

Several connections were independently shown to be modulated between conditions in both cohorts (controls and patients): ITG to SPL, SPL to vlPFC, and dlPFC, vlPFC to dlPFC, and the intrinsic connections in V1, vlPFC, and dlPFC. After finding the set of extrinsic and intrinsic connections that changed between MSIT conditions, we fitted the winning model (N22 for controls; N26 for patients) to each participant. Example model predictions for each of the three populations are shown in Supplementary Figure 8. Blue and red lines correspond to the two MSIT conditions (control and interference). There are three pairs of lines, corresponding to the three populations of the JR model (cf. Figure 1, right panel). After fitting the model, we obtained connections (*A, B*) and other biophysical parameter estimates from each participant (*G, H, T*; see Methods). These were used as DCM predictors in the next section.

### Classification

**FIGURE 3:**
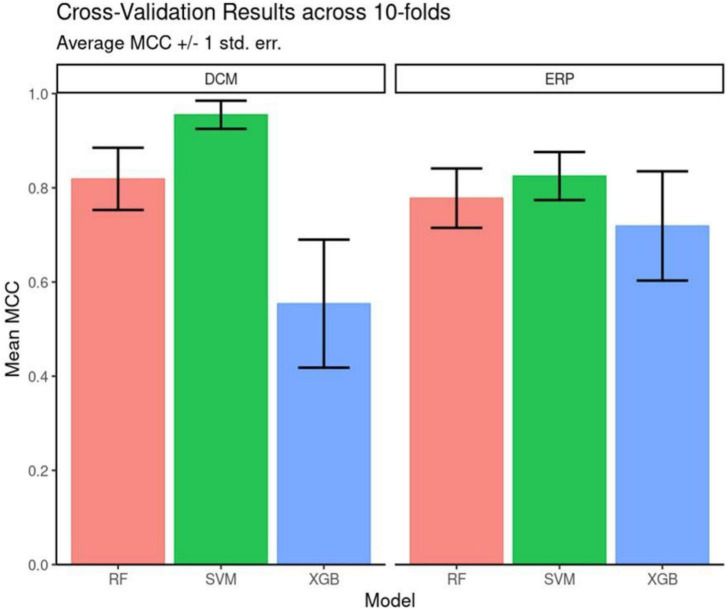
Cross validation performance. Matthew’s Correlation Coefficient scores across the 10-fold cross-validation. Mean and +/– 1 standard error are reported for each model architecture. Support vector machines (SVM) classifiers performed the best for both feature sets on average across the 10 folds.

Our goal was to assess whether DCM features can better measure depression’s internal heterogeneity, compared to EEG features. To do this, we asked whether DCM features achieved significantly better classification than EEG features (56). EEG features included ERP parameters, i.e., ERP peak amplitude and latency differences between the two MSIT conditions. We used ERPs from 24 channels so that the number of EEG predictors was equal to the number of DCM predictors. This allowed us to perform head-to-head comparisons between ERP and DCM predictors. Crucially, we chose ERP predictors in such a way that it biases subsequent analysis against DCM predictors (they were the best classifiers in an initial random forest model; see Methods for more details). Due to the small sample (15 patients), it was not possible to test out of sample predictions for algorithm robustness. To assess the relative ability of DCM and ERP parameters to capture diagnostic heterogeneity (and thus support better classification), we computed classification performance using three algorithms: SVM, Gradient Boosted Tree and Random Forest (Figure 3). The SVM algorithm performed best for both the DCM and ERP data sets (highest average MCC scores).

Overall, DCM features led to better classification accuracy than EEG features across all three algorithms tested. SVM was used for assessing feature importance because it had the highest classification performance (see Supplementary Table 3 for full results of MCC and F-Score). It also had a more parsimonious hyperparameter set (two–kernel gamma and cost) compared to either decision-tree ensemble model.

**FIGURE 4:**
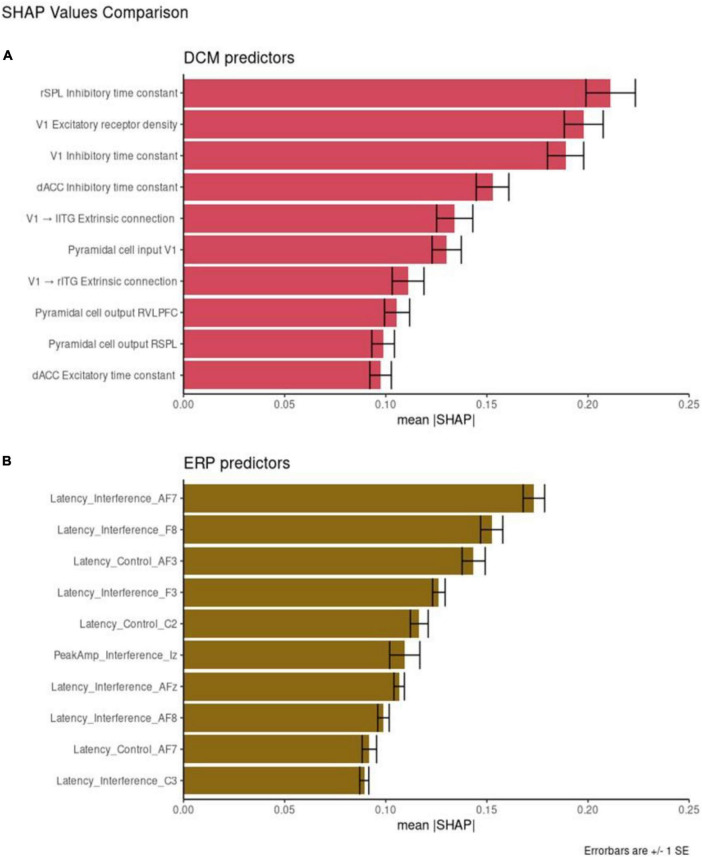
Feature importance. Shapley additive explanation (SHAP) values for the 10 best performing dynamic causal modeling (DCM) features **(A)** and event-related potentials (ERP) features **(B)**. Mean absolute SHAP values are reported with +/– 1 SE.

To evaluate feature importance, we used absolute SHAP values averaged across participants. Figure 4 shows SHAP values for the 10 most important DCM (Figure 4A) and EEG (Figure 4B) features. By taking the average across each of the 49 classification decisions (participants), we have a relative rank of feature importance. Overall, the DCM features have higher mean absolute SHAP values compared to EEG features.

Individual DCM features have higher SHAP values that the EEG features with the same corresponding rank. For example, the most important DCM predictor is the rSPL inhibitory time constant (mean SHAP = 0.22). The most important ERP predictor is AF7 Latency score in the interference condition (mean SHAP = 0.17). These results are directly comparable, with the larger SHAP score reflecting greater feature importance.

We also compared the mean SHAP scores between the two datasets, with DCM scoring higher than ERP across all ten most important features (the SHAP value of the top DCM feature is larger than the corresponding SHAP value for the top ERP feature; the SHAP value of the second-best DCM feature is larger than the corresponding SHAP value for the second-best ERP feature, etc.). We compared the mean absolute SHAP values in a paired *t*-test using rank as the pairwise grouping. The 10 top DCM features had higher SHAP values than the 10 top EEG features [*t* = 4.28, *p* = 0.002, CI = (0.01, 0.03)].

**FIGURE 5:**
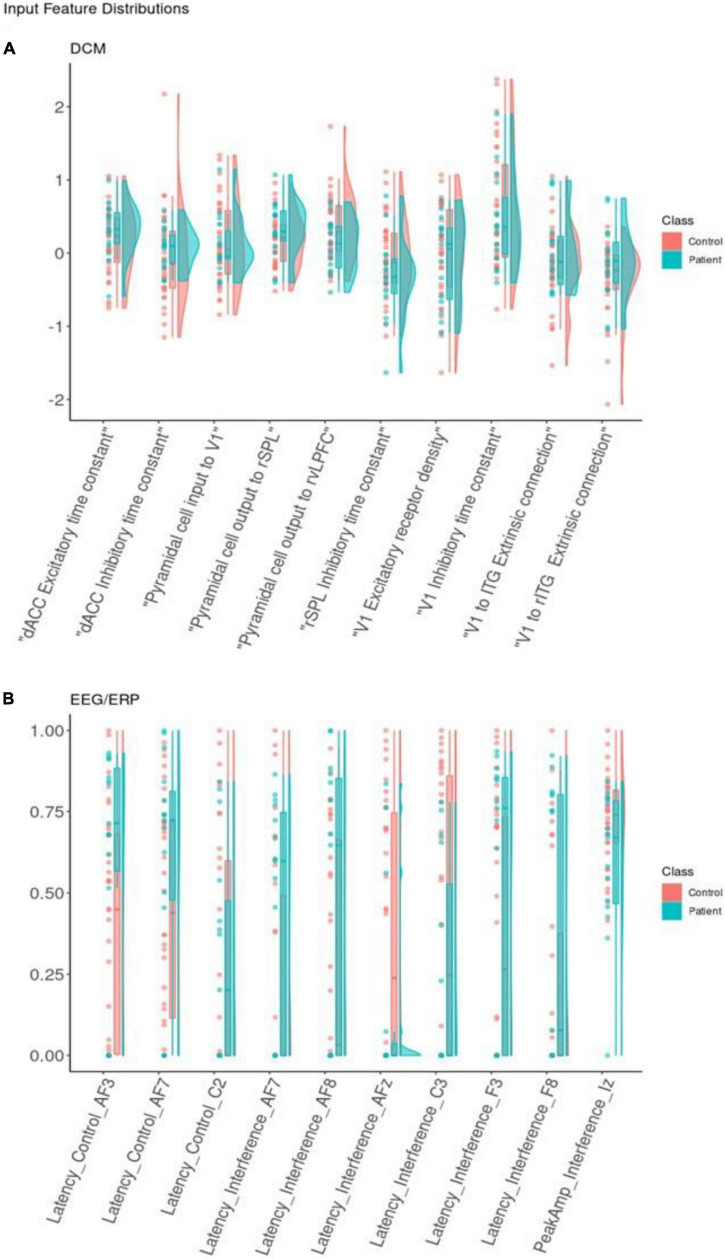
Feature distribution. Distributions for the 10 best dynamic causal modeling (DCM) input features **(A)** and (electroencephalogram/event-related potentials) (EEG/ERP) input features **(B)**. Classification features represented using dots for real observations alongside boxplots and density curves to show the shape of the distribution. Each is colored by class. Each input feature on the x-axis is shown with raw data points, boxplots, and density curves. Patients (blue) and controls (red) are shown separately. The EEG/ERP features are shown after min-max scaling to present latency and interference variables together on a comparable scale.

Overall, DCM features appeared equivalent or more powerful than EEG features (higher SHAP values). They also captured separate subtypes of depressed participants better. This may relate to DCM features’ ability to capture variability between individuals. Figure 5 shows the distributions of the ten most important DCM and EEG features. Visually, DCM features show distributions with central tendencies, with areas of non-overlap between patient and control distributions. This can be explained by the Laplace approximation used to define the posterior densities of DCM parameters (66). They occupy less of the available numeric range. ERP features are more uniformly dispersed over the available range.

### Unsupervised clustering

**FIGURE 6:**
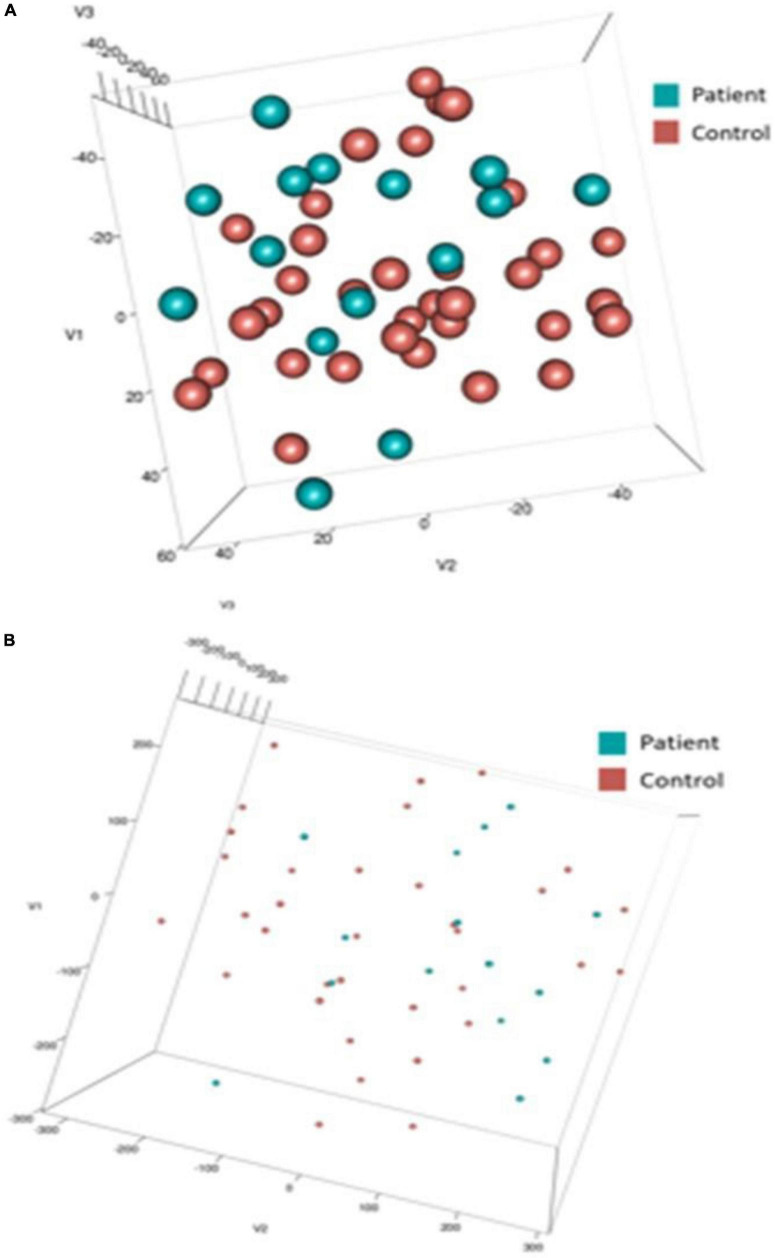
t-SNE embeddings. Three dimensional t-SNE values for top 10 input features of dynamic causal modeling (DCM) **(A)** and electroencephalogram/event-related potentials (EEG/ERP) **(B)**. *Post-hoc*, data points were colored blue (Patient) and red (Control). Note the difference in point size reflects the difference in axis scale (–/+ 40 in DCM compared with –/+ 300 in EEG).

To capture depressive heterogeneity, t-SNE representations were made from unlabeled data of each of the 10 most important features for DCM and ERP feature sets. *t-*SNEs were generated using a perplexity value of 25 over 2,500 iterations. These embeddings were labeled *post hoc* to determine if these features could elucidate differences between patients and controls. Figure 6 shows the three dimensional representations of patient embeddings in blue and control embeddings in red. Supplementary Figure 10 shows the t-SNE results for two dimensional t-SNE representations. DCM feature embeddings show clustering tendencies, while EEG feature embeddings were more dispersed throughout the lower dimensional spaces with no clear patterns.

**FIGURE 7:**
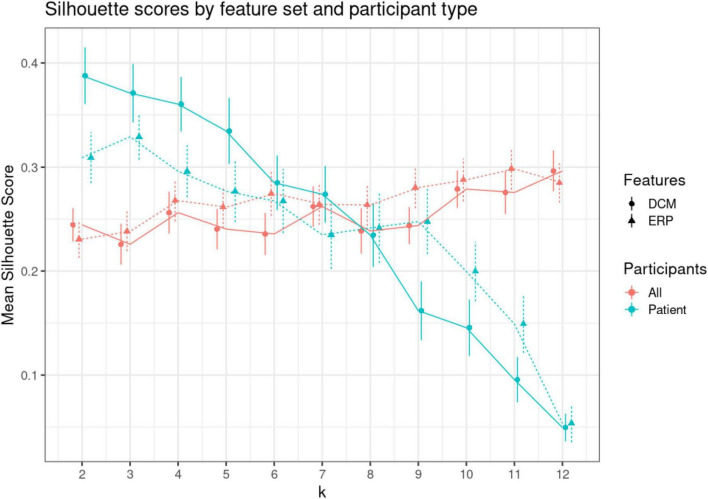
*K*-means clustering. Clustering was performed using varying numbers of *k* (from 2 to 12) to cluster (1) all participants, (2) only patients. To find the optimal *k*, we used Silhouette scores. Silhouette scores quantify the ratio of within over between cluster distance. A positive value signifies appropriate clustering (74). This figure shows the mean Silhouette scores (vertical axis) for each level of *k* (shown on the horizontal axis). Red dots show scores for patients and controls (all), while green dots show scores for patients only. Solid lines and discs depict scores for dynamic causal modeling (DCM) features, while dashed lines and triangles for electroencephalogram (EEG) features. Error bars show +/– 1 SE.

Our patient dataset included two depression subtypes, bipolar, and unspecified depression. We asked whether we could recover this using the t-SNE spaces obtained using DCM and EEG biomarkers. We clustered the t-SNE embeddings using k-means and compared Silhouette scores for all participants and for the patient cohort only. Figure 7 shows the mean Silhouette score with ± 1 standard error. Scores for both DCM and EEG results across 2–12 clusters (*k*) can be found in Supplementary Table 4. Silhouette scores decrease monotonically, suggesting that a two cluster representation is the most parsimonious solution in this small dataset. The lack of change in Silhouette scores over increasing values of *k* for the all-participants dataset suggests that there is no clear clustering solution that separates, e.g., controls and two or more patient types. This may reflect unstructured heterogeneity in the control participants.

The DCM embeddings for the patients only at low *k* values had the highest mean Silhouette scores [μ = 0.388, CI = (0.334, 0.441)]. Compared to the second highest Silhouette score, ERP embeddings for patients only, the DCM embeddings were significantly higher when compared using a two-sided *t*-test (*t* = 2.17, *p* = 0.032). Interestingly, DCM Silhouette scores decreased in a monotonic fashion (blue solid line in Figure 7), thus confirming the two known clusters in the patient dataset.

## Discussion

We demonstrated a proof of concept that transforming electrophysiological data to underlying biophysical parameters using DCM may reliably capture variability that correlates with clinical status. Although our dataset is small and heterogeneous, DCM features were equivalent to or outperformed raw EEG features on most classification metrics, and this held true across multiple classifier algorithms. Our results align with prior work that has successfully used DCM to identify differences between unipolar and bipolar depression (22). We considered participant-specific (first-level) analysis and group effects (second-level analysis) as two separate steps. An alternative approach could be to combine these steps into a single hierarchical model (35, 66). We will consider this elsewhere. Similarly, we used a Fixed Effects approach that could be replaced by Mixed-Effects (7, 8).

If our DCM-based approach can be replicated on a larger dataset, it may provide an intriguing avenue for personalized medicine. There is a growing set of TMS and similar tools for manipulating brain connectivity (67, 68). For any individual patient, the approach we describe permits the identification of which DCM feature(s) are driving that patient’s vulnerability. Although this is still a speculative claim, it may be possible to then target and normalize those specific features. The feasibility of that approach will depend on whether these features change as a patient undergoes treatment or remain present even during euthymia (69, 70).

We found that MSIT-induced variance was explained by feedforward connectivity from primary sensory and object discrimination areas to prefrontal cortex, plus changes in intrinsic, within-region connectivity. This is consistent with current working models of cognitive control, the construct tested by MSIT. In those models, information about cognitive control demands is computed posteriorly, then fed forward to anterior structures (dlPFC which then influences motor circuits) (71, 72). Given that we explored a wide range of connectivity changes, our data-driven recovery of a known phenomenon provides some confidence that we identified known effects. At the same time, our analyses were based on prior assumptions about anatomically plausible connections. In future work, we will test these assumptions using BMS.

Dynamic causal modeling (DCM) suggested a low dimensional space of potential biomarkers (we went from 240 EEG-based to 92 biophysical features). This helps fitting prediction models on limited datasets. As an alternative to PCA (69, 73), DCM-derived parameters are also directly interpretable; e.g., DCM synaptic connectivity or intrinsic excitability can directly map to potential treatments (20). Another DCM advantage is that its biophysical models provide ways to test potential explanations of pathophysiology. This advantage can also be a limitation: for example, some prior knowledge about cortical regions and their interactions is required. Finally, we note that DCM is not simply a biophysical model. It also involves source reconstruction (i.e., analyses in source space) while the ERP analyses are in sensor space. This source attribution adds further explainability, and may explain why the DCM parameters had higher explanatory power on this specific dataset.

We here used a small patient dataset, to present a proof of concept that biophysical biomarkers are a feasible approach to depression subtyping. That dataset is inherently limited, and our current results should not be treated as generalizable. In future work, we will analyze larger patient datasets, which are increasingly available for secondary analysis (74). We will address data leakage by using a hold-out test set of data observations to evaluate classification model performance. This will allow for better comparisons between model architectures and depression subtyping using self-supervised learning algorithms like TABnet (75).

We emphasize that the classification performance of our models should not be treated as a claim that the current pipeline carries diagnostic or clinical utility. The sample size is too small; we and others have pointed out that small-sample-derived biomarkers frequently do not generalize (14, 76, 77). Although we did perform some internal cross-validation, this sample size did not permit us to follow best-practices for preventing data leakage (78). Specifically, we performed SMOTE up-sampling on the full dataset, before conducting a training/validation split. On the other hand, the goal of this work was not to develop a reliable classifier and report its performance. The classification approach was used solely to compare the relative performance of DCM vs. ERP features (similar susceptibility to data leakage and effect size inflation). Regarding the ERP features themselves, we only found statistically significant differences in ERP latencies, not peak amplitudes. We could have used average amplitudes within an epoch, but this would not change our results, as the average and peak amplitude were highly correlated (*R* = 0.98 for the control and *R* = 0.92 for the interference condition). Related to this, a significant difference between DCM and the standard ERP technique is that the ERP focuses on two specific aspects of the polyphasic ERP response (peak amplitude and peak latency). By virtue of its model fitting, DCM considers the full shape/temporal evolution of the ERP. One approach to addressing this might be to perform a principal component analysis (PCA) or similar decomposition on the ERPs. One could then compare, e.g., eigenvalues of the first few PCs against the DCM derived features. In this proof of concept study, we focused on alignment with earlier work (5–8). We used the most common ERP markers, i.e., peak amplitude and latency. A more detailed exploration of potential ERP features will be considered elsewhere.

Our feature importance scoring (SHAP) results should also be interpreted with caution. With this caveat, over half the highly weighted features from the DCM analysis came from more caudal regions and included within region features. This aligns with recent results in a larger dataset, where signals in primary sensory regions were more able to classify (non)response to antidepressant treatment than were signals from higher-order cognitive/associative regions (79). On the other hand, those response predictors were generally unstable in a cross-validation analysis (80). In all, we do not make claims that our specific identified markers/clusters are new generalizable findings. Rather, they are proof of concept for a larger point: that DCM parameters can be scored and interpreted, and that subtypes might be identifiable by clustering. With a larger dataset, it would become feasible to identify robust DCM features (78). There are such recent datasets available (81, 82), particularly in depression (51, 74). In future work we will consider these new datasets together with additional covariates, like stressful life events (83), biological sex (6), and others. These can easily be combined with the DCM pipeline we have shown here.

Another caveat is that t-SNE does not preserve distances and global structure in the data. It only preserves local geometry while constructing the low dimensional embedding (84). Thus one might wonder whether it is an appropriate tool for discovering clusters in biomarker data. Indeed, we did not find unknown clusters that might reflect phenotypic or other differences. Our only point was that using DCM—not ERP—biomarkers projected onto t-SNE spaces allowed us to recover the known patient clusters in our data, bipolar and unspecified depression. This is similar to the use of the t-SNE algorithm in bioinformatics (85, 86), where t-SNE has been used to obtain hierarchical clusters and recover clusters obtained with different methods, like genetic analyses. t-SNE extensions are also useful for discovering unknown structure. There are also recent t-SNE algorithms that can address the above geometry distortion, like parametric (87) and hierarchical SNE (88). We will consider them in future work.

In summary, we have demonstrated the first proof of concept for a novel approach to identifying psychiatric biomarkers from EEG, based on converting manifest EEG signals to interpretable biophysical parameters. We demonstrated the viability of this approach against the same biomarker pipeline applied to manifest data (in this case, ERPs). If applied to larger datasets and a more robust variety of data sources, this DCM-based pipeline can be an important new approach to dissecting the heterogeneity of depression and depressive vulnerability.

## Data availability statement

Publicly available datasets were analyzed in this study. This data can be found here: https://drive.google.com/drive/folders/1X5_NJJDqyV11va5asOdLHMPski-zrJ1a?usp=sharing and https://github.com/pinotsislab/ERP-Feature-Extraction-and-Classification/tree/main.

## Ethics statement

The studies involving human participants were reviewed and approved by Massachusetts General Hospital Institutional Review Board. The patients/participants provided their written informed consent to participate in this study.

## Author contributions

DAP and ASW: conceptualization, methodology, funding acquisition, and resources. DAP and SF: writing—original draft. All authors validation, formal analysis, investigation, data curation, writing—review and editing, and visualization.

## Abbreviations

DCM: Dynamic Causal Modeling
ERP: Event-related Potentials
MSIT: Multi-Source Interference Task
JR: Jansen and Rit mass model
ICA: Independent Component Analysis
MCC: Matthew’s Correlation Coefficient
SHAP: Shapley Additive Explanation
PCA: Principal Component Analysis.
