# Supplementary material for: Toward biophysical markers of depression vulnerability
Source: Front Psychiatry. 2022 Oct 18;13:938694. doi: 10.3389/fpsyt.2022.938694 (PMC9622949; doi:10.3389/fpsyt.2022.938694)
Supplement: Supplementary file 1 [file Data_Sheet_1.DOCX]

# Supplementary Methods

## Dataset and Task

The dataset includedwas generated from 15 psychiatric patients who reported current or past depressive symptomsand 34 non-diagnosed controls. Importantly, this dataset was not limited to patients diagnosed with unipolar depression, but included bipolar and unspecified depression. We considered this a better demonstration of our approach to heterogeneity. This is a secondary analysis of a cohort collected in a previous study^28^. No additional human participants review was required for this secondary analysis, as the primary study’s data had been deposited in an online repository (<https://transformdbs.partners.org/>), from which we obtained de-identified data.As this repository is no longer functional at the time of publication, we have provided a copy of the dataset, see below.All participants gave informed consent for the primary study, which was overseen by the Massachusetts General Hospital Institutional Review Board.

EEGs were recorded with a 70-channel electrode cap, based on based on the 10–10 electrode-placement system (Easycap, Vectorview, Elekta-Neuromag, Helsinki, Finland), using a sampling rate of 1000 Hz. All recordings were completed in an electro-magnetically shielded room, with individual channel impedances kept below 5000 Ohm.

EEGs were collected as participants performed theMulti-Source Interference Task (MSIT).^29^ The task requires participants to report on a presented stimulus by using their index, middle or ring finger to press three buttons corresponding to numbers 1, 2, and 3 respectively. The stimulus appears on a screen displaying three numbers; one number (the target) is different from the other two (distractors). The participant identifies the target by pressing the corresponding buttons. There are two task conditions, control and interference, which are interleaved to prevent development of response sets. During control trials, the distractor numbers are zeros and the location of the target number is aligned with its corresponding button. In interference trials, the distractors are non-zero numbers (valid targets; flanker effect), and the target is in a location misaligned with that of the button (Simon effect).

The dataset was provided in a pre-processed state whereby artefacts had been removed using Independent Component Analysis (ICA) and bad trials (e.g., excessive rates of voltage change suggesting artefact) removed.These analyses had been performed through visual inspection of individual ICs and channels by trained operators. Information was not available on the exact number of rejections per subject.The continuous signal data had been segmented into epochs using a time window of 1.5 seconds before stimulus onset and 2 seconds after. No further artefact rejection was performed in this study.Aligned post stimulus epochs were used to extract Positive Potential components (see below).

## ERP Analysis

Positive Potential components were extracted from 70 EEG channels (average ERPs over participants are included in Figure 1A; see also Suppl. Figure 1). Previous MSIT studies found differences in the Positive Potential component between task conditions.^32, 33^Later-occuring Positive Potentials are a common signature of conflict and cognitive control, and are believed to arise from mid-frontalprocessing of incongruent stimuli.^34,35^Data provided included artefact rejection and initial filtering. We cut trials/epochs and usedstimulus-locked analyses based on the Positive Potential signal. This was defined as evoked responses between 250ms - 350ms after event onset. Only trials with correct responses were used.We did not baseline-correct the ERPs, as this data was not provided as part of the publicly available dataset. We considered trial averages and identified Peak Latency, Peak Amplitude, Mean Amplitude (called Mean Peak) for each channel. Peaks were identified using Matlab’sfindpeak function. Wilcoxon tests for the effect of conditions (interference vs. control), on each channel across controls and patients showed that latency (but not peak amplitude) exhibited significant differences, seeSuppl. Table 1. *P*-values were corrected for multiple comparisons using a Bonferroni corrected threshold value of *p<*0.0007.

## Dynamic Causal Modelling (DCM)

We used Dynamic Causal Modelling (DCM)^20-22,36-41^, which allows one to infer processes at the neuronal level from scalp EEG measurements^2^. DCM models the changes ofintrinsic (within area)and extrinsic (between area) connections across task conditions. It allows one to assess whether information flow changes in the same way (top-down,bottom-up or both) between the two task conditions across all participants. DCM includes a biophysical model that predicts the neuronal activity that underlies the observed EEG signal.^42^This model comprises a Jansen and Rit (JR) neural mass that included three populations of neurons: excitatory pyramidal cells, excitatory spiny stellate cells, and inhibitory interneurons (smooth stellate cells). These populations are connected with one anotherfor excitatory (black) and inhibitory (red) connections, and also with populations in other areas (Figure 1B). JR models can predict both evoked and induced responses and have been used in theoretical and experimental studies.^27, 43-46^DCM was implemented using SPM12.

Beyond DCM, the JR model has been used to describe complex brain dynamics including epilepsy^47^ and TMS effects.^48^ It was the first model of a cortical circuit that produced a variety of brain dynamics (evoked responses, attractor states, spike-wave discharges etc.) by simply varying its parameters. In the context of DCM, the JR model has been used to predict ERPs during attention and other cognitive tasks, including Mismatch Negativity (MMN).^49,25,50^

DCM also includes an observation model that transforms neuronal activity predictions from the biophysical model above to a predicted scalp-observed EEG signal. We here used the JR model and DCM to fit ERPs during the MSIT. Our analysis focused on time domain data. The observation model for each brain area corresponds to an equivalent current dipole (ECD).^51^ There are as many dipoles as brain areas. Each dipole has 6 parameters: 3 for its location, 2 for its orientation and 1 for each amplitude. For more details, see ^52^. By estimating the parameters of both models simultaneously, we can separate different sources of variability in the EEG signal like neuronal dynamics, volume conduction and other sources of noise observed with EEG. Fitting exploits a non-linear optimization (Bayesian) approach.^32^ Briefly, neuronal dynamics are prescribed by the JR model. Then they are projected to the EEG sensor level via an ECD model. Both models (JR and ECD) are included in DCM. Their parameters are estimated simultaneously to account for conditional dependencies. Because DCM estimates both the observation and neural models, it effectively performs source reconstruction from EEG electrodes to neural sources. Their parameters are estimated simultaneously to account for conditional dependencies.

DCM was implemented using SPM12. The functional network modelled with DCM can be seen in Figure2 (cf. model M1 in top left corner, all other models include the same network and assume changes in different connections, explained below). This network is comprisedof areas activated during the MSIT.^29, 53^ Changes in functional connectivity within this network were observed, at the group level, in patients with depression.^54-56, 42^The following anatomical regions were selected (in MNI coordinates)^35^: primary visual cortex (V1) [24, -83, 7], right inferior temporal gyrus (rITG) [52, -54, -14], left inferior temporal gyrus (lITG) [-54, -33, -25], right superior parietal lobule (rSPL) [23, -59, 56], left superior parietal lobule (lSPL) [-33, -58, 57], right ventrolateral prefrontal cortex (rVLPFC) [44, 25, -12], left ventrolateral prefrontal cortex (lVLPFC) [-44, 35, -5], right dorsolateral prefrontal cortex (rDLPFC) [35, 41, 18], left dorsolateral prefrontal cortex (lDLPFC) [-28, 48, 4] and dorsal anterior cingulate cortex (dACC) [2, 3, 53]. We did not model other medial cortical structures (e.g., dorsomedial PFC or supplementary motor area) because it was not clear that their activity could be reliably disambiguated from underlying dACC. We similarly combined midline structures where left/right disambiguation would be uncertain. We considered a parsimonious brain network that included areas from each level of the cortical hierarchy – sensory, temporal, parietal, dorsal and ventral frontal areas, and ACC. Our assumption was that ERPs resulted from coordinated activity across this hierarchy. EEG recordings reveal their temporal profile, which, in turn, is the result of different temporal scales characterizing dynamics in each brain area. This affects signal propagation in different parts of the network and might be different in participants whose depressive symptoms arise from different network dysfunctions. Such differences, if they existed, can be revealed by DCM and the machine learning algorithms used below. This is because DCM estimates neural parameters after accounting for the observation model.

The biophysical model included in DCM predicts Positive Potential responses. Data on the other hand, included ERP recordings from different participants, patients and controls. By fitting the DCM model predictions to this data, the variability in ERP recordings results in variability in the biophysical model parameter estimates across participants. That is, apparent noise or heterogeneity in the scalp-level recordings might arise from a small number of disruptions in the underlying network, which could be considered as biotypes or endophenotypes of depression.

Because we did not know how connectivity changed between task conditions, we compared several variants of the same biophysical model describing the network of Supplementary Figure 2. We considered a network containing all of our modelled brain regions: V1, ITG, SPL,vlPFC, dlPFC and dACC. We assumed forward and backward connections between specific areas, as well as lateral connections between homologous areas in the right and left hemispheres. The model variants differed in the connectionsthat could vary between the two task conditions. Following Pinotsis, Buschmann, and Miller^40^, we first considered changes ofextrinsic connections (i.e. between nodes) only. The first twenty candidate models we considered are shown in Supplementary Figure 2. There were 12 extrinsic connections that could change between conditions. We assumed that these could change between one or more neighbouring pairs of brain areas: occipital to parietal, parietal to frontal and frontal to dACC, that is, V1🡪 ITG, ITG🡪SPL, SPL 🡪{vlPFC, dlPFC} and vlPFC🡪 dACC. Connections that were permitted to change in each model are depicted with red arrows. Feedforward connections are depicted with bottom-up arrows, feedback connections with top-down arrows. Assuming that only the forward connections changed between MSIT conditions, we obtained models M1-M10 in Supplementary Figure 2. Only bottom-up arrows are red. Assuming that only backward conditions changed, we then obtained models M11-M20. Here, only top-down arrows are red. Similarly, assuming that both forward and backward connections changed, we obtained another ten models. These include ten models similar to those in Supplementary Figure 2, where both bottom-up and top-down arrows are red. The remaining fifteen models include models where we relaxed the constraint that between-condition differences would only be reflected in connections between neighbouring pairs of brain areas. in the first five models, we assumed that feedforward connections between V1🡪 ITG changed along with one of the five core connections,ITG🡪SPL, SPL 🡪vlPFC, SPL 🡪dlLPFC, vlPFC🡪 dlPFC and vlPFC🡪 dACC. The next five models were similar, where instead of feedforward we considered feedback connections. The last five models included assumptions that both feedforward and feedback connections changed. Overall, the candidate model space comprised 45 models. To sum up, we compared all candidate models where forward or backward connections changed between different parts of the brain network. This yielded the extrinsic connections that were modulated during the task. After comparing these 45 models, we found one that could explain the data best (had the highest evidence). Then, we considered variations of this model assuming changes in intrinsic connections from each node to itself (in addition to changes in extrinsic connections that the winning model above assumed). We thus assumed that intrinsic connections could change at any (combination of) brain areas: V1, ITG, SPL, {vlPFC, dlPFC} and dACC. For example, assuming that intrinsic connections changed in only one of the above five brain areas (the two PFC subareas considered together), we obtained models N1-N5 in Supplementary Figure 3. The remaining models are just all possible combinations of the above five models. Thus, we compared 32 candidate models in total.

## Bayesian Model Selection (BMS)

For model comparison, we used an approach known as Bayesian model selection (BMS). This was performed assuming fixed-effects (FFX).^57^BMS fits competing models to EEG data and assesses the most likely model. This exploits a Bayesian cost function (called relative Free Energy^33^), which quantifies model evidence (i.e., the Free Energy is a score of model fits). The Free Energy is similar to the Bayesian Information Criterion, BIC, in that it includes a complexity term dependent on both the number of parameters and their posterior correlations. That is, models with more free parameters are automatically penalized compared to models with fewer. Then, the most likely model (among a set of candidate models) is the one with the largest Free Energy. This is quantified in terms of the Bayes Factor (BF). This is a Bayesian analogue of the usual odds ratio and quantifies the probability that one out of many models has generated the EEG data. It provides the (relative) probability that this could be a true model of the brain compared to alternative models. Using BF, we can pool together evidence of the most likely model across different participants.BF is defined as the ratio of the Free Energy of two models. If BF*(1vs2)*>3, we can say that model 1 is better than 2—or more exactly, there is strong evidence for 1 relative to 2, see^40^. Note that the Free Energy can be thought of as an alternative to cross-validation in terms of preventing overfitting. Cross-validationassesses how well a model can explain unseen data, while Free Energy yields the most parsimonious model to explain observed data by penalizing for complexity.^58^These two processes have been shown to be mathematically equivalent.^59^

## Feature Sets

Two distinct data sets of input features were used to train machine learning models, DCM parameters and EEG features. DCM parameters from individual subjects were obtained after fitting the biophysical model to EEG data, see previous section. Recall that the model included 12 extrinsic connections in each hemisphere connecting 10 brain areas in total. Each of these areas included a microcircuit like the one shown in Figure 1B. This includes 10 excitatory and inhibitory connections, each characterized by a synaptic time constant and synaptic efficacy, and recurrent connectivity parameters characterizing the gain of pyramidal cells and inhibitory interneurons. To sum up, the biophysical model included the following parameters identified for each participant: extrinsic connectivity, *A* (12x2=24 parameters)*,* differences in extrinsic connectivity between MSIT conditions, *B*(24 parameters, derived from the model fitting as shown in Results)*,* excitatory and inhibitory receptor density, *G* (2x10=20 parameters)*,* strength of connections between the three populations of the JR model shown in Figure 1B, *H* (4 parameters; see arrows in Figure 1B)*,* and excitatory and inhibitory synaptic time constants, *T*(20 parameters). The above parameters were used as predictor features in machine learning algorithms below. We thus obtained92DCM predictor features. The DCM features used included intrinsic and extrinsic connections that were found to differ between MSIT conditions in both the patient and control DCM fits.

To compare the predictive power of the DCM parameter estimates against the EEG features, we used an equal number of ERP features.The full set of potential EEG features included 240 variables (60 EEG Channels x 2 conditions x 2 variables, i.e. ERP peak amplitude and latency differences between the two MSIT conditions). We reduced the number of channels to 24 so that the total number of EEG features was similar to the number of DCM parameters to reduce bias when comparing ERP and DCM feature sets. We performed permutation testing to select the channels of greatest information gain.^60, 61^The ERP features were chosen based on their contribution toa random forest model (constructed without hyperparameter tuning). This “naive” random forest allowed us to select channels with features that were most beneficial in separating classes while still allowing for multiple interaction effects between features. The tradeoff of this method stems from using a reduced number of features with the benefit that they are potentially more meaningful, and easier to interpret. The selected channels are included in Supplementary Table 2.

## SMOTE Oversampling and Model Training

Out of the 49 participants, only 15 were patients with depressive symptoms. The dataset was thus imbalanced between control and patient classes, which can affect supervised and unsupervised classification. We implemented an over-sampling approach known as Synthetic Minority Over-sampling Technique (SMOTE) to correct for this imbalance.^62^Oversampling was performed using the Themis library in R [65]. This calculates the *k*-nearest neighbors for each observation from the minority class and creates synthetic examples along line segments joining the observations with random nearest neighbors. It has successfully been applied to datasets including electronic health records [63] and image segmentation [64], demonstrating improvements in overall model accuracy. We used SMOTE for supervised classification to augment an imbalanced dataset. SMOTE improves the sensitivity of models tested at the cost of diluting the information of biomarkers in real world sampling. Sub-clusters of depression (subtypes in the minority class) cannot be found using data augmented with SMOTE because the method inherently creates artificial clusters. Synthetic observations are created with values near real observations of the minority class. SMOTE was used with *k*= 3 nearest neighbors to generate data for 19 synthetic patients, resulting in 68 observations total (49 real participants and 19 synthetic observations). This brought the classes to parity.The dataset used for training and tuning included 34 controls and 34 patients. See Supplementary Figure 9 for a visual depiction of the sampling strategy. In Supplementary Figure 9A we depict the augmented dataset created by using SMOTE oversampling. In panel B, we depict the 10 fold cross-validation that was used for model training and hyperparameter tuning.10 folds of the data are constructed, with each withholding a separate 1/10th of the data for evaluating the model accuracy. This resulted in a stratified sample of the classes (each fold contained an equal number of class observations).

We used the Tidymodels ecosystem in R for training[65].Model hyperparameter tuning was performed using grid search. Fifty hyperparameter combinations were assessed based on a Latin hypercube assignment. Hyperparameters were chosen based on the model with the highest average Matthew’s Correlation Coefficient (MCC) score across the withheld 10% folds.This method was applied to both the ERP and DCM training data sets separately.

Cross-validation was used to train classifiers and assess whether DCM features can better measure depression’s internal heterogeneity, compared to EEG features.^66^ We compared multiple performance metrics, including*F*-measure (aka F1-score), and the Matthews’ Correlation Coefficient (MCC).^69^ MCC is easier to interpret compared to Cohen’s Kappa, as there is no reference to the expectation of class size, which may not be known and has advantages over the F-measure with regards to class imbalance in binary classification.^70^ Similarly, although hyperparameter search/tuning should usually be conducted on a validation set that is completely separate from training and test sets^71^, here we are comparing the same number of DCM and EEG features, with the same model structure and hyperparameter tuning. Model fitting is only being used to evaluate DCM and ERP’s relative accuracy.

## Feature Importance

The best performing classifier was selected by mean MCC score across all ten folds. This classification algorithm wasthen used to compute feature importance scores. Shapley additive explanation (SHAP) values were constructed to assess feature importance. SHAP values predictions on the original data set (49 participants, no SMOTE augmentation).^72^SHAP values were constructed using subsampling of the different combinations of the input features and attributing a weight representing how much credit features should receive for class prediction. SHAP values quantify the importance of a feature based on classification accuracy independently and in conjunction with all possible subsets of features involving the feature of interest (SHAP values arise from scoring the effect of “coalitions” of features). This reveals how efficient the low dimensional space spanned by DCM and EEG features is in describing the internal heterogeneity of patients with depressive symptoms.

In general, SHAP values weight predictive power of features. Feature importance across the entire sample of observations is assessed by adding the prediction weights for all classes.Note that SHAP values can be positive or negative in binary classification (contributing or detracting from a positive classification), therefore absolute SHAP values were used.The predictive power of EEG features vs. DCM features was compared directly because the corresponding SHAP values take on the same scale and are predicting the same underlying data.

## Unsupervised Clustering

The ten most important features as determined by SHAP values from both the ERP and DCM feature sets were used to construct embedding scores with t-stochastic neighbor embeddings (*t-*SNEs). *t-*SNEs are useful for visualizing and exploring higher-dimensional data in lower dimensional representations.*t-*SNEs are scores used for dimensionality reduction when non-linear relationships exist in data.^73^ The method iteratively reduces the KL divergence between a Gaussian probability distribution of similarity between data points in high dimensional space and a *t-*distributed representation in lower dimensional space. Here, we used three dimensions as the lower dimensional space. This provided visualizations of the data that were convenient for assessing subtypes or clusters of patients with depressive symptoms.

Clustering was performed using *k-*means in the three dimensional space obtained by *t-*SNE. *K*-means is a method of unsupervised machine learning that iteratively groups bundles of observations to reduce within-cluster sum squares distances and increase the sum squared distance between cluster centroids.^74, 75^. *K*-means depends on an *a priori* number of clusters. The optimal cluster number can be found by computing Silhouette scores across all candidate values of *k*. Observations which been classified appropriately have a lower mean distance between points within their assigned cluster compared to the mean distance to points in the next-nearest cluster neighbors.^76^ This ratio is given by Silhouette scores. We computed the Silhouette score averaged over all data features (in the low dimensional *t-*SNE space) for all candidate values of *k* from two to twelve.
